# Rare Variants in *NOD1* Associated with Carotid Bifurcation Intima-Media Thickness in Dominican Republic Families

**DOI:** 10.1371/journal.pone.0167202

**Published:** 2016-12-09

**Authors:** Nicole D. Dueker, Ashley Beecham, Liyong Wang, Susan H. Blanton, Shengru Guo, Tatjana Rundek, Ralph L. Sacco

**Affiliations:** 1 John P. Hussman Institute for Human Genomics, University of Miami, Miami, Florida, United States of America; 2 Dr. John T. Macdonald Foundation Department of Human Genetics, University of Miami, Miami, Florida, United States of America; 3 Department of Neurology, Miller School of Medicine, University of Miami, Miami, Florida, United States of America; 4 Department of Public Health Sciences, Miller School of Medicine, University of Miami, Miami, Florida, United States of America; Central South University, CHINA

## Abstract

Cardiovascular disorders including ischemic stroke (IS) and myocardial infarction (MI) are heritable; however, few replicated loci have been identified. One strategy to identify loci influencing these complex disorders is to study subclinical phenotypes, such as carotid bifurcation intima-media thickness (bIMT). We have previously shown bIMT to be heritable and found evidence for linkage and association with common variants on chromosome 7p for bIMT. In this study, we aimed to characterize contributions of rare variants (RVs) in 7p to bIMT. To achieve this aim, we sequenced the 1 LOD unit down region on 7p in nine extended families from the Dominican Republic (DR) with strong evidence for linkage to bIMT. We then performed the family-based sequence kernel association test (famSKAT) on genes within the 7p region. Analyses were restricted to single nucleotide variants (SNVs) with population based minor allele frequency (MAF) <5%. We first analyzed all exonic RVs and then the subset of only non-synonymous RVs. There were 68 genes in our analyses. Nucleotide-binding oligomerization domain (*NOD1)* was the most significantly associated gene when analyzing exonic RVs (famSKAT p = 9.2x10^-4^; number of SNVs = 14). We achieved suggestive replication of *NOD1* in an independent sample of twelve extended families from the DR (p = 0.055). Our study provides suggestive statistical evidence for a role of rare variants in *NOD1* in bIMT. Studies in mice have shown Nod1 to play a role in heart function and atherosclerosis, providing biologic plausibility for a role in bIMT thus making *NOD1* an excellent bIMT candidate.

## Introduction

Cardiovascular disorders including myocardial infarction [MI] and ischemic stroke [IS] are leading causes of death and disability in the US[1]. Risk factors for these disorders include atherosclerosis, diabetes and smoking[1]. Heritability studies have implicated a role for genetic factors in both MI and IS; however, identified loci explain only a fraction of the heritability[2]. One approach to identify genetic variants influencing complex traits such as MI and IS is to study intermediate phenotypes which are hypothesized to be closer to the underlying biology and less complex than the disease end-product. Total carotid intima-media thickness [IMT], a subclinical measure of atherosclerosis[3], is one intermediate phenotype for these disorders.

IMT measures the thickness of the carotid artery wall, and increased IMT is associated with greater risk for IS[4,5] and MI[6,7]. Total IMT is composed of three different intima-media thickness measurements; measurements at the common carotid, bifurcation [bIMT], and internal carotid artery. In previous studies, we and others have shown total IMT and its individual components to be heritable, with heritability estimates between 0.3 and 0.6 in several populations[8–11]. The underlying biology of each component is believed to differ, and therefore, might be expected to have different genetic etiologies[12]. Several candidate regions and individual SNPs have been implicated in total IMT and its individual components through linkage[8,13,14] and association studies[15–21], including several from our own family-based studies of IMT in extended families from the Dominican Republic[8,15,16].

Taking a multi-stage approach to identify genetic signals influencing IMT, we first performed a genome-wide linkage study on several measures of IMT, including bIMT, which revealed a region on 7p to be linked with carotid bIMT[8]. We then fine-mapped this signal in two stages. First, we genotyped tag SNPs within the linkage region and found common variants in *ANLN* and *AOAH* to be associated with bIMT[15]. We then re-sequenced *ANLN* and *AOAH* to fully characterize the genetic architecture underlying the associations at these genes and found rare variants [RVs] in *ANLN* to be associated with bIMT as well[16]. While our re-sequencing study allowed us to perform an in-depth characterization of *ANLN* and *AOAH*, we have shown that our most significantly associated variants explained some, but not all, of the linkage signal[15]. Therefore, for our current study we expanded our investigations to encompass the rest of the 7p region to identify additional variants contributing to the linkage signal. We hypothesized that additional RVs in this region are associated with bIMT. Indeed, our candidate gene re-sequencing study, as well as other recent studies, have shown that sequencing regions under linkage peaks can successfully identify RVs influencing complex traits and disorders[16,22–25], particularly in extended families. Extended families are well-suited for the identification of RVs for several reasons including: 1) Pedigrees may be enriched for individual RVs, making them easier to study[26,27] and 2) Co-segregation of an RV with the trait can be examined to help differentiate between causal and non-causal RVs[26,27].

Therefore, we expanded on our previous studies and re-sequenced the rest of the linkage peak on 7p in nine extended families from the Dominican Republic to better characterize the genetic variation in this region and to identify additional RVs accounting for this signal.

## Materials and Methods

### Study Samples

To identify RVs associated with bIMT we utilized data from both NOMAS [the Northern Manhattan Study], a population based cohort, and the Family Study of Stroke Risk and Carotid Atherosclerosis, a family study consisting of select probands from NOMAS and their family members[28]. Details of NOMAS and the family study have been published previously[28,29]. Briefly, NOMAS is a population-based cohort study of stroke risk and cognitive decline. A total of 3,298 stroke-free participants were enrolled in NOMAS from 1993–2001. A subset of participants were enrolled in the family study; these were Caribbean Hispanic probands from NOMAS with a high risk of cardiovascular disease[29] who could provide a family history, obtain family members’ permission for research staff to contact them and have at least three first-degree relatives able to participate. All probands were identified in northern Manhattan and family members were enrolled in New York at Columbia University and in the Dominican Republic [DR] at the Clinicas Corazones Unidos in Santo Domingo. All participants provided written informed consent and the study was approved by the Institutional Review Boards of Columbia University, University of Miami, the National Bioethics Committee, and the Independent Ethics Committee of Instituto Oncologico Regional del Cibao in the DR.

A total of 100 Dominican families were enrolled in the family study, of which, twenty-one families with a family-specific LOD score >0.1 at the chromosome 7p QTL for bIMT were selected for our current study; nine families were included in our discovery analyses (n = 153 individuals) and twelve families were included in our replication analyses (n = 164 individuals). Our discovery families were chosen from a large targeted sequencing study which sequenced the 1 LOD down region under linkage peaks for six traits in 20 families; total IMT, bIMT, left ventricular mass, plaque presence, plaque area, and left atrial size. Families were selected for sequencing if they had family-specific LOD score ≥ 0.4 for any trait. A total of five families met this criteria for bIMT and were included in the discovery sample for our current study. Additionally, four families were sequenced that had family-specific LOD score >0.1 for bIMT and were also included in our discovery sample (Table 1). All remaining families with family-specific LOD score >0.1 for bIMT that were not sequenced were genotyped on the customized Illumina Exome Array and included in our replication sample (n = 12 families). Additional replication analyses were performed using a sample of 561 unrelated Dominicans from the NOMAS cohort study[28] who were not enrolled in the family study. These individuals were also genotyped using the customized Illumina Exome Array. To facilitate confirmation of variants identified via sequencing, all individuals included in our discovery sample were also genotyped on the customized Illumina Exome Array.

**Table 1:** Characteristics of Dominican families included in analyses for chr7 analyses.

Family ID	Individuals per Family	Family-Specific LOD Score	bIMT Residual	
			Min	Max
*Discovery families*				
5987	15	0.81	-2.21	3.25
253	12	0.5	-1.53	3.05
5103	33	0.48	-1.36	1.38
803	11	0.47	-1.9	1.66
4965	10	0.42	-1.19	1.12
3630	13	0.33	-1.16	1.94
3631	16	0.15	-1.39	1.09
4623	10	0.15	-2.85	1.79
5569	16	0.11	-1.47	1.1
*Replication families*				
4099	11	0.31	-1.86	0.36
5501	15	0.27	-1.34	1.27
5699	17	0.27	-0.5	3.04
4613	6	0.2	-1.4	2.47
289	10	0.18	-0.75	0.87
4541	15	0.17	-0.79	0.44
921	21	0.14	-1.04	1.02
3695	14	0.14	-1.42	1.29
2009	11	0.13	-0.95	1.83
1431	19	0.12	-2.56	2.08
2133	15	0.11	-0.8	1.47
357	10	0.11	-1.89	0.82

### Carotid and Associated Risk Factor Measurements

As described in detail previously[15,30], high-resolution B-mode carotid ultrasound was performed according to the standardized and validated scanning and reading protocols. Carotid IMT was measured outside the areas of plaque as recommended by consensus documents[5] using an automated computerized edge tracking software M’Ath (Intelligence in Medical Technologies, Inc., Paris, France). Carotid bifurcation was defined as a segment beginning at the tip of the flow divider and extending 10 mm proximal to the common carotid artery. Carotid bifurcation IMT (bIMT) was calculated as a composite measure of an average of the means of the near and the far walls of carotid bifurcation IMT in the both sides of the neck. Our cIMT measurements have excellent consistency as published previously[28].

Vascular risk factors, including dyslipidemia, hypertension, diabetes, BMI, waist to hip ratio (WHR), and pack-years of smoking, were collected during a standardized interview[31]. Hypertension was defined as self-reported history of high blood pressure, systolic blood pressure ≥ 140 mmHg, diastolic blood pressure ≥ 90 mmHg, or self-reported use of antihypertensive medication. Diabetes was defined as self-reported history of diabetes mellitus or fasting glucose level ≥ 126 mg/dL. Dyslipidemia was defined as a self-reported history of hyperlipidemia or total cholesterol > 240 mg/dL. BMI was defined as weight (kg) divided by height (m)^2^ and WHR was defined as waist circumference divided by hip circumference. Pack-years of smoking were calculated as the number of cigarettes smoked per day divided twenty and then multiplied by the number of years smoking.

### Sequencing and Quality Control

Genomic DNA was isolated from whole blood. Targeted sequencing of the exons in 68 genes within the 1 LOD unit down region on 7p (chr7:29.3Mb-42.8Mb) was performed using a customized Agilent SureSelect Enrichment kit. A detailed description of sequencing methods has been previously published[16]. Briefly, DNA libraries were sequenced on an Illumina HiSeq2000 and the raw sequencing reads were aligned to the human reference sequence hg19 with the Burrows-Wheeler Aligner (BWA)[32]. Variant calling was performed using the Genome Analysis ToolKit (GATK) and potential functions of variants were annotated using ANNOVAR (http://annovar.openbioinformatics.org/) and SeattleSeq (http://snp.gs.washington.edu/SeattleSeqAnnotation138/index.jsp).

Quality control was conducted in accordance with procedures we published previously[15]. Briefly, variants with VQSLOD < -4 or call rate < 75% were removed and within each individual sample, variants with a depth < 4 or Phred-Like (PL) score < 100 were set as missing. Novel variants in genes with nominal evidence for replication were validated via Sanger sequencing. At the sample level, concordance between the sequencing data and genotypes from the previous peak-wide association study[15] were assessed for each sample. Two samples with low concordance (<95%) were removed. Additionally, for our current study, individuals missing bIMT measures (n = 3) and/or covariate values (n = 10) were removed. For the remaining family study samples, pedigree structure was confirmed using the Graphical Relationship Representation software. Mendelian error checking was performed and Mendelian errors were set to missing for all the variants called using PLATO[33]. We additionally removed non-exonic variants, leaving us with 136 samples from 9 families and 791 exonic RVs available for analyses.

### Exome Array Genotyping and Quality Control

All participants were genotyped using the Illumina HumanExome-24v1_B Beadchip, containing 551,076 SNVs, at the Hussman Institute for Human Genomics in the Center for Genome Technology (Miami, FL, USA). Our Exome Array included custom content of approximately 6,000 exonic variants, selected on the basis of sequencing data obtained in the discovery family data set. Variants were selected if they: 1) were not included on the Illumina HumanExome-12v1_B Beadchip; 2) passed sequencing quality control in at least three individuals; 2) could not be effectively imputed using Affymetrix 6.0 GWAS markers (imputation quality score <0.8); and 3) had an Illumina Infinium^®^ design score > = 0.6. Genotype calling was performed using Illumina’s GenTrain version 1.0 clustering algorithm in GenomeStudio version 2011.1 and a GenCall cutoff score of 0.15 was used. The data was re-clustered within this sample set and then cluster plots for 13,476 SNVs were manually examined using Illumina’s suggested protocol. This resulted in the manual re-clustering of 8,949 SNVs and removal of 376 SNVs leaving a total of 550,700 SNVs for quality control analyses, including 2,493 SNVs within our 7p region. Genotypes for the samples were then extracted and quality control procedures performed.

Of the 1,029 participants genotyped on the Exome array (n = 151 discovery family members, 180 replication family members, 698 NOMAS participants), 97.3% had a genotype call rate > 98% (n = 1,001). Part of Exome Chip genotyped samples also had targeted sequencing data and/or Affymetrix 6.0 whole-genome genotype data available. Using overlapping variants found across the Affymetrix 6.0 genotyping chip (n = 61,118 SNVs) and targeted sequencing data (n = 8,969 SNVs), we calculated sample concordance values. All samples had high concordance with whole-genome genotype data and/or targeted sequencing data (≥96%). We removed 21 individuals due to unexpected duplicates, gender discrepancy and unexpected relatedness, and low call rate (<98%). For each variant found in the family data sets, Mendelian error checking was performed using PLATO and all errors were set to missing.

For our current study, individuals missing bIMT measures and/or covariate values were removed (n = 11 family members and 98 NOMAS participants), leaving us with a final sample of 137 family members within 9 discovery families, 164 family members within 12 replication families and 561 Dominican NOMAS participants. At the variant level, we removed variants with call rate<95% (n = 1 in the families, n = 2 in NOMAS), concordance < 98% with the sequencing data (n = 11 variants), monomorphic SNVs and non-exonic SNVs. This left us with 508 exonic RVs in our region for Exome Array analysis.

### Statistical Methods

#### bIMT Association Analyses

As in previous analyses, bIMT was natural log transformed to ensure a normal distribution[8,15,16]. Rare variants were defined based on frequencies from Dominican NOMAS participants, as described previously[16]. Variants were classified as rare if they had MAF<5% or could not be imputed efficiently (INFO ≤0.4) in NOMAS Dominicans. Association analyses were performed using targeted sequencing data in nine Dominican families (discovery families). Using this data, we performed gene-based analyses with the Family SNP-set (Sequence) Kernel Association Test (Fam-SKAT)[34], adjusting for age, age^2^, sex, age*sex, pack years of smoking, waist hip ratio (WHR), and body mass index (BMI). Covariates were identified using a polygenic screen implemented in SOLAR, with covariates having p<0.1 included in analyses. Analyses were restricted to genes with ≥ 2 polymorphic variants. We employed two different gene-based analyses based on annotation from ANNOVAR and SeattleSeq: exonic RVs (UTR3, UTR5, synonymous, missense, nonsense, splice-site variants) and a subset of non-synonymous RVs only (missense, nonsense or splice-site variants). A p<0.00037 was considered significant based on a Bonferroni correction of 136 tests (68 genes with ≥ 2 RVs analyzed under two approaches).

As in our previous analysis[15], we used SAS to compute the residual bifurcation value after adjusting for the covariates included in our analyses in order to examine the distribution of RVs in relation to bIMT. Additionally, we performed single-variant association analyses for all variants within our most significantly associated gene in order to explore this association further. The Quantitative Transmission-Disequilibrium Test (QTDT) was used for these analyses. QTDT tests were implemented in SOLAR and adjustment was made for the same covariates included in our gene-based analyses.

#### Replication Analyses

Replication analyses were performed using Exome array data to test RVs in the 68 genes captured in the targeted sequencing for association with bIMT in two different samples; 1) Twelve additional Dominican families consisting of 164 individuals, and 2) 561 Dominican NOMAS participants. Prior to analyses, bIMT was natural log transformed. Gene-based association analyses were performed using Fam-SKAT for the replication families and SKAT-O for the NOMAS participants, adjusting for the same covariates included in our discovery analyses. Additional adjustment was made for the first principal component and presence of hypertension in the NOMAS participants, as these covariates were associated with bIMT in this sample (p<0.1). These analyses were performed using the same two sets of variants analyzed in the discovery analyses; all exonic RVs and then a subset of non-synonymous RVs only. Analyses were restricted to genes with ≥ 2 polymorphic variants.

Similar to our discovery families, the residual bifurcation value was calculated in our replication families after adjusting for associated risk factors using SAS. This was done to better visualize the distribution of RVs in relation to bIMT. We also performed single variant association analyses for our most significantly associated gene in both replication samples, adjusting for the same covariates included in our gene-based analyses. The QTDT, implemented in SOLAR, was performed in the replication families, and linear regression, implemented in PLINK[35], was performed in the NOMAS sample. For linear regression analyses, variants were coded additively.

## Results

### Participant and SNP Characteristics

A total of 136 individuals in nine families from the DR were available for our discovery analyses. These families had family-specific LOD scores that ranged from 0.11 to 0.81 for bIMT in the chromosome 7p region. Residual bIMT values ranged from -2.85 to 3.25 (Table 1). Within these nine families, sequencing identified 791 exonic RVs, of which 17.2% (n = 136) were either missense, nonsense or splice site variants (Table 2). Of the 791 RVs identified, 288 were found on the customized Exome Array and polymorphic in our replication samples.

**Table 2:** Polymorphic variants identified by re-sequencing of 7p region in 9 discovery families.

Variant Function†	Total	MAF*	
		<1%	1%<5%
Exonic	450	273	177
Splice Site	4	4	0
UTR 3’ or UTR 5’	337	196	141
*Total*	*791*	*473*	*318*

*MAF based on NOMAS DR Frequencies

^†^Function based on ANNOVAR annotation

Family-specific LOD scores for our replication families were generally lower than those for our discovery families, with LOD scores ranging from 0.11 to 0.31. Residual bIMT values ranged from -2.56 to 3.04 (Table 1). Within these replication families, 274 polymorphic exonic RVs were detected with 35.8% being missense, nonsense or splice site variants (n = 98) (Table 3). Over half of the missense, nonsense or splice site RVs in our replication families were also polymorphic in our discovery families (n = 55). In the NOMAS sample, 489 polymorphic exonic RVs were detected with 44% (n = 216) being missense, nonsense or splice site variants (Table 3).

**Table 3:** Polymorphic Exome Array variants passing QC in the 7p region in the 12 replication families and the NOMAS DR sample.

Variant Function†	Total	MAF*	
		<1%	1%<5%
***Replication Families***			
Exonic	152	84	68
Splice Site	1	1	0
UTR 3’ or UTR 5’	121	58	63
*Total*	*274*	*144*	*131*
***NOMAS DR***			
Exonic	312	241	71
Splice Site	2	2	0
UTR 3’ or UTR 5’	175	106	69
*Total*	*489*	*349*	*140*

*MAF based on NOMAS DR Frequencies

^†^Function based on ANNOVAR annotation

### bIMT Association Results

Using our nine Dominican families with sequencing data, we performed gene-based analyses testing RVs in 68 genes across the 7p region for association with bIMT in two ways: 1) Analyzing all exonic RVs, and 2) Restricting analyses to non-synonymous RVs. Eleven genes were nominally associated with bIMT in at least one of these analyses (p<0.05) (Table 4). For analyses involving all exonic RVs, nucleotide-binding oligomerization domain 1 (*NOD1*) was our most significantly associated gene (p = 9.16x10^-4^). This association became attenuated when analyses were restricted to non-synonymous RVs (p = 0.07). In our replication families, *NOD1* achieved suggestive significance in both our exonic (p = 0.055) and non-synonymous (p = 0.07) analyses. In NOMAS, *NOD1* was suggestively associated when analyzing non-synonymous RVs (p = 0.06).

**Table 4:** Gene-based association results for rare variant analyses in the chr 7p region, for genes with p<0.05 in at least one analysis.

Gene	CM Start	Discovery Families				Replication Families				NOMAS			
		Exonic RVs*		Non-Synonymous RVs*		Exonic RVs*		Non-Synonymous RVs*		Exonic RVs*		Non-Synonymous RVs*	
		# SNVs	p	# SNVs	p	# SNVs	p	# SNVs	p	# SNVs	p	# SNVs	p
*NOD1*	48.7	14	**9.16 x 10**^**−4**^	4	0.06	9	0.06	5	0.07	18	0.19	10	0.06
*LOC646999*	60.1	13	**0.004**	--	--	--	--	--	--	--	--	--	--
*CCDC129*	51.0	11	**0.004**	7	**0.02**	4	0.45	2	0.33	23	0.79	19	0.64
*AQP1*	50.3	17	**0.006**	--	--	5	0.06	2	0.25	11	0.70	5	0.23
*PPP1R17*	51.2	12	**0.02**	2	0.09	2	0.37	--	--	4	0.36	1	0.23
*EPDR1*	57.8	13	**0.02**	4	**0.02**	5	0.26	2	0.31	6	0.17	2	0.49
*VPS41*	59.4	19	**0.04**	3	**0.002**	3	0.66	--	--	11	0.25	5	0.40
*LINC00265*	50.2	30	**0.04**	--	--	--	--	--	--	--	--	--	--
*ZNRF2P1*	52.2	8	**0.04**	--	--	--	--	--	--	--	--	--	--
*EEPD1*	55.5	25	**0.05**	2	0.31	9	0.11	--	--	18	0.48	3	0.82
*NME8*	57.7	8	0.06	4	**0.04**	5	0.44	5	0.44	7	1.00	7	1.00

*RV = rare variant

**Fig 1:**
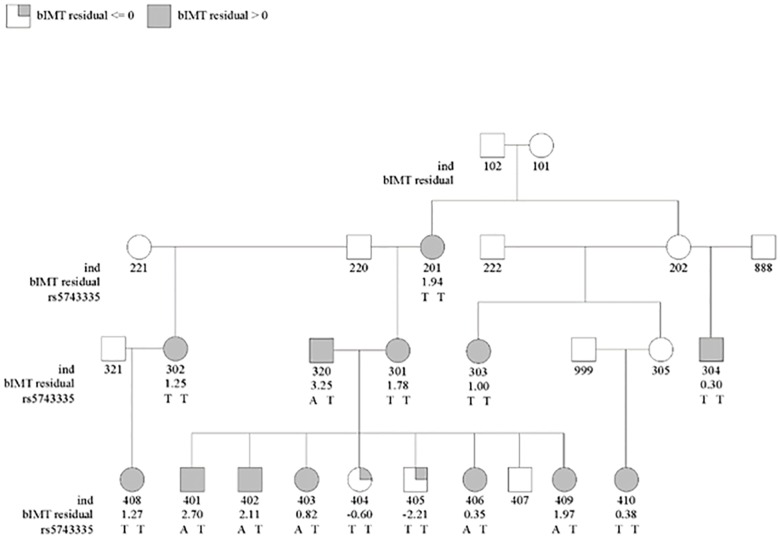
Pedigree of Family 5987 depicting residual bIMT value and rs5743335 genotype, our most significantly associated variant in *NOD1*. Individuals with residual bIMT value ≤ 0 are shaded gray in the upper right quadrant and individuals with residual bIMT value > 0 are shaded completely gray.

To explore the *NOD1* association further, we performed single-SNP association analyses for all RVs included in the gene-based analyses and found two SNPs to be associated with bIMT in the discovery families: rs5743335 (p = 5.08x10^-4^) and rs112070346 (p = 0.04) (Table 5). Our most significantly associated SNP, rs5743335, was observed in six individuals within Family 5987, all of whom had positive bIMT residuals (Fig 1). This variant was also observed once in two other families (Family 3630 and Family 5569), with both individuals also having positive bIMT residuals. Interestingly, rs5743335 was associated in the replication families as well, although the direction of effect differed. In the discovery families, individuals with the TA genotype had higher mean residual bIMT values than individuals with the TT genotype (1.58 v. -0.08). The opposite was seen in the replication families (-0.79 v. 0.02). This variant was not associated in NOMAS, although individuals with the TA genotype had a slightly higher mean bIMT value than individuals with the TT genotype (ß = 0.02). The other associated variant, rs112070346, was not associated in the replication families or NOMAS.

**Table 5:** Single-SNP association results for rare variants in *NOD1*.

SNP	BP	Minor /Major Allele	Discovery Families			Replication Families			NOMAS			Function
			MAF	Direction of Effect*	p-value†	MAF	Direction of Effect*	p-value†	MAF	Direction of Effect*	p-value†	
rs5743335	30498829	A/T	0.03	+	**5.08x10**^**-4**^	0.02	-	**0.01**	0.01	+	0.56	UTR5
rs112070346	30464932	G/T	0.02	-	**0.04**	0.02	-	1	0.004	+	0.56	UTR3
rs78206797	30498842	G/A	0.004	+	0.11	0.003	+	0.75	0.002	-	0.86	UTR5
rs140817833	30486592	A/G	0.02	+	0.12	0.003	+	1	0.004	+	0.81	missense
rs145135608	30494794	A/G	0.01	+	0.12	--	--	--	0	--	--	missense
rs2975634	30491693	T/C	0.10	+	0.14	0.02	-	0.83	0.04	+	0.21	missense
rs3020208	30492142	A/G	0.10	+	0.14	--	--	--	--	--	--	synonymous
rs5743343	30491698	T/C	0.008	-	0.51	0.006	-	0.55	0.002	+	0.66	synonymous
rs5743341	30492190	T/C	0.02	+	0.63	--	--	--	0.007	-	0.08	synonymous
rs6960726	30496625	T/C	0.004	-	0.99	--	--	--	--	--	--	UTR5
rs5743374	30464872	A/G	0.004	+	1	--	--	--	--	--	--	UTR3
rs370080093	30491668	A/G	0.004	-	1	--	--	--	--	--	--	synonymous
rs5743342	30491919	T/C	0.004	-	1	0.05	+	0.23	0.02	-	0.86	missense
rs5743340	30492598	A/C	0.004	+	1	--	--	--	--	--	--	synonymous
rs138669564	30465123	G/A	--	--	--	--	--	--	0.004	-	0.29	UTR3
7_30486645	30486645	A/G	--	--	--	--	--	--	0.003	+	0.26	synonymous
rs151170709	30491081	A/G	--	--	--	0.02	-	0.85	0.003	-	0.6	missense
rs5743347	30491123	T/C	--	--	--	--	--	--	0.003	+	0.12	missense
rs5743345	30491220	A/G	--	--	--	--	--	--	0.004	+	**0.01**	missense
rs147561327	30491915	T/C	--	--	--	--	--	--	0.002	+	0.53	missense
rs145823866	30492017	A/G	--	--	--	0.02	+	0.23	0	--	--	missense
rs62636578	30492202	A/G	--	--	--	--	--	--	0.004	+	0.6	synonymous
rs144535612	30492392	T/C	--	--	--	--	--	--	0.003	-	0.28	missense
rs141934741	30492468	T/C	--	--	--	--	--	--	0.003	+	**0.008**	missense
rs140176390	30492599	G/A	--	--	--	--	--	--	0.0009	-+	0.97	missense

*+ indicates increased bIMT,—indicates decreased bIMT

^†^QTDT for families; linear regression for NOMAS

While *NOD1* was the most significantly associated gene when analyzing all exonic RVs, *VPS41* was our most significantly associated gene in the non-synonymous RV analysis (p = 0.002). This gene was not associated in the NOMAS dataset (p = 0.40) and replication was not possible in the replication families because no polymorphic SNVs were observed. Of the remaining 9 genes with p<0.05 in at least one analysis, one gene, *AQP1*, achieved nominal replication in the replication families (Table 4). No remaining genes achieved nominal replication in the NOMAS sample.

## Discussion

Using a multi-stage approach, culminating in the targeted sequencing of a linkage region identified through previous analyses[8,15,16], we have identified *NOD1* to be a possible candidate for carotid bifurcation IMT. In our gene-based analysis of exonic RVs, we found *NOD1* to be the most strongly associated gene in our nine extended families from the Dominican Republic. Exonic RVs in *NOD1* also showed suggestive association in our replication sample of twelve extended families from the Dominican Republic and non-synonymous RVs were suggestively associated in an additional replication sample from the Dominican Republic. These findings suggest a role for *NOD1* in bIMT in our extended families from the Dominican Republic and possibly the general Dominican population as well. Though the association did not reach conventional significance (p = .055) in our replication families, it is important to note the limited sample size and therefore, diminished power to detect significant association. Additionally, these families had weaker evidence for linkage to bIMT in this region than the nine sequenced families and therefore a weaker signal would be expected in these families relative to the discovery families. Furthermore, our replication analyses were limited to only those variants found on the customized Exome Array, which contained only nine of the fourteen exonic RVs discovered through targeted sequencing in the discovery families. Therefore it is possible that associated variants in *NOD1* may have been missed.

When characterizing the *NOD1* association further by examining individual variants within the gene, we identified several candidate variants. The most strongly associated single variant was rs5743335 which is found in the 5’ UTR region of *NOD1* and is suggested to be located within a chromatin enhancer in HUVEC Umbilical Vein Endothelial Primary Cells, according to haploreg[36]. This variant was associated with bIMT in both the discovery and replication families, albeit with opposite effects. Carriers of the A allele of rs5743335 in the discovery families had higher bIMT residuals, compared to non-carriers and a similar trend was seen in the NOMAS sample, although this finding was not significant. In contrast, carriers of the A allele of rs5743335 in the replication families tended to have lower bIMT residuals compared to non-carriers. It may be that this SNV is in linkage disequilibrium with the causative variant. Alternatively, this could have occurred as a result of rs5743335 interacting differently with other variants or the environment in the replication families relative to the discovery families. Lastly, it could suggest a false positive finding. Further investigation is warranted.

Since rs5743335 was not associated with bIMT in the NOMAS sample, this could reflect our lack of statistical power to detect this association, or it could indicate that this association does not extend to the general Dominican population, and may only be observed in the genetic and environmental context defined by our selected families. Indeed, to be enrolled in the family study, the proband had to have a high risk for CVD and, for this study, we restricted our analyses to only include families with strong evidence for linkage in the chromosome 7p region. Therefore, rs5743335 may only be associated with bIMT in this setting.

*NOD1* encodes the nucleotide-binding oligomerization domain containing protein 1, a member of the nucleotide-binding oligomerization domain-like receptors (NLRs)[37]. NOD1 is found in the cytosol and recognizes peptides from bacterial peptidoglycan and damaged or abnormal host cells. NOD1 is known to play a role in the innate immune response as well as inflammatory responses making it an excellent candidate for bIMT[37,38]. Activation of NOD1 causes NF-kB activation, cytokine production, and apoptosis induction[37]. Interestingly, our previous analyses identified *AOAH* to be a candidate gene for bIMT[15], another gene with a known role in innate immunity. *AOAH* encodes a lipase that deactivates lipopolysaccharide on Gram-negative bacteria which then activates toll like receptor [TLR] signaling[39]. TLRs and NLRs are both pattern recognition receptors which work to detect pathogens as part of our innate immune system[40–42]. Therefore, perhaps it is through their effects on the innate immune system that our previously identified candidate genes and *NOD1* influence bIMT.

Studies in mice have revealed Nod1 to have a role in heart function and atherosclerosis, thus providing further biologic plausibility for NOD1’s role in bIMT. One study found Nod1 to be expressed in the heart and administration of a Nod1 agonist for two weeks led to fibrosis, cardiomyocyte apoptosis and cardiac dysfunction, as measured by decreased ejection fraction, collagen deposition and higher expression of type I collagen[43]. Another study found that chronic administration of selective Nod1 ligands induced coronary arteritis and valvulitis[44].

Additionally, one study found that when Nod1 ligand FK565 was administered to apolipoprotein E [Apoe] knockout mice, development of atherosclerosis was accelerated. Further, in *Apoe*^*-/-*^ and *Nod1*^*-/-*^ double-knockout mice, atherosclerotic lesion area in aortic root was reduced compared to *Apoe*^*-/-*^ knockout mice suggesting a role for Nod1 in atherosclerosis[45]. In humans, a previous study found the *NOD1* rs5743336 polymorphism to be significantly more common in stroke patients seropositive for Chlamydia pneumonia compared to controls seropositive for Chlamydia pneumonia[46]. The *NOD1* rs5743336 polymorphism is a common variant that was observed in our discovery families. Interestingly, while this variant was not associated with bIMT (p = 0.33), it was in complete LD with our top SNP, rs5743335 (D’ = 1, r^2^ = 0), with the rare allele of rs5743335 always found with the common allele of rs5743336. Together, these studies support the biologic plausibility for a role of *NOD1* in bIMT.

An additional candidate gene identified through this study was *AQP1*. Rare variants in *AQP1* were nominally associated in our discovery families (p = 0.006) and replication families (p = 0.06). *AQP1* encodes the aquaporin-1 protein which is expressed in endothelial cells, including those lining atherosclerotic plaques[47]. A study in rats showed that this protein is present during vascular development and following vascular injury. It was additionally shown that aquaporin-1 is necessary for water transport across human vascular smooth muscle cells[47]. Aquaporin-1 has been shown to promote angiogenesis[48] and may be involved in salt-sensitive hypertension[49].

Our study has several limitations. First, replication analyses were performed using Exome Array data, thereby limiting our analyses to only those variants included on the Exome Array. However, variants on the Exome Array were selected to be functional (primarily missense variants) and had to be observed at least three times in at least two people (http://genome.sph.umich.edu/wiki/Exome_Chip_Design). Second, our study was limited to individuals from the Dominican Republic and may not be generalizable to other populations. Third, due to our sample sizes, we had limited power to detect RVs in our replication samples.

In conclusion, this study, in combination with our previous studies investigating the chromosome 7p region, highlights the complexity of the genetic underpinnings of bIMT and how a multifaceted approach is necessary to identify genetic candidates. Our investigation of rare variants identified *NOD1* to be a strong candidate for bIMT, a gene previously not associated when analyzing common variants.

## Supporting Information

S1 Phenotype File Phenotype data for study participants. (CSV)

S1 Pedigree File Pedigree file for discovery and replication families. (CSV)

S1 Map File PLINK.map file for discovery family data included in analyses. (MAP)

S1 Ped File PLINK.ped file for discovery family data included in analyses. (PED)

S2 Map File PLINK.map file for replication family data included in analyses. (MAP)

S2 Ped File PLINK.ped file for replication family data included in analyses. (PED)

S3 Map File PLINK.map file for NOMAS data included in analyses. (MAP)

S3 Ped File PLINK.ped file for NOMAS data included in analyses. (PED)
